# Epithelial Transfer of the Tyrosine Kinase Inhibitors Erlotinib, Gefitinib, Afatinib, Crizotinib, Sorafenib, Sunitinib, and Dasatinib: Implications for Clinical Resistance

**DOI:** 10.3390/cancers12113322

**Published:** 2020-11-10

**Authors:** Richard J. Honeywell, Ietje Kathmann, Elisa Giovannetti, Carmelo Tibaldi, Egbert F. Smit, Maria N. Rovithi, Henk M.W. Verheul, Godefridus J. Peters

**Affiliations:** 1 Department of Medical Oncology, Amsterdam UMC, VU University Medical Center, P.O. Box 7057, 1007 MB Amsterdam, The Netherlands; r.honeywell@amsterdamumc.nl (R.J.H.); i.kathmann@vumc.nl (I.K.); e.giovannetti@amsterdamumc.nl (E.G.); mn.rovith@vumc.nl (M.N.R.); 2 Department of Pharmacy, Amsterdam UMC, VU University Medical Center, P.O. Box 7057, 1007 MB Amsterdam, The Netherlands; 3 Cancer Pharmacology Lab, AIRC Start-Up Unit, Fondazione Pisana per la Scienza, 56017 Pisa, Italy; 4 Division of Oncology, Department of Oncology, S. Luca Hospital, 55100 Lucca, Italy; carmelo.tibaldi@uslnordovest.toscana.it; 5 Department of Thoracic Oncology, Netherlands Cancer Institute, 1066 CX Amsterdam, The Netherlands; e.smit@nki.nl; 6 Department of Medical Oncology, Radboud University Medical Center, Geert Grooteplein Zuid 8, 6525 GA Nijmegen, The Netherlands; henk.verheul@radboudumc.nl; 7 Department of Biochemistry, Medical University of Gdansk, 80-211 Gdansk, Poland

**Keywords:** tyrosine kinase inhibitors, CaCo2 model system, gut-epithelial transfer, efflux ratio, red blood cell accumulation

## Abstract

**Simple Summary:**

Tyrosine kinase inhibitors (TKIs) specifically inhibit phosphorylation of signaling pathways of cancer cells, thereby inhibiting their growth. They are characterized by a poor solubility and high protein binding, leading to a large variability in gut uptake after oral administration and variation in the clinical efficacy. We used the CaCo2 gut epithelial model to characterize the gut absorption of 7 TKIs and observed a large variation in apical/basolateral (mimicking gut/blood) transfer, with 4 TKIs showing a negative and 3 a neutral transfer. A highly negative transfer may lead to pharmacokinetic resistance. Intracellular uptake of TKIs was high for sunitinib and crizotinib, intermediate for gefitinib, dasatinib and sorafenib, low for afatinib and not detectable for erlotinib. These properties may explain a high red blood cell to plasma ratio for most TKIs investigated. Although TKIs are poorly absorbed the latter property may compensate for this.

**Abstract:**

*Background:* tyrosine kinase inhibitors (TKIs) inhibit phosphorylation of signaling proteins. TKIs often show large variations in the clinic due to poor pharmacology, possibly leading to resistance. We compared gut absorption of inhibitors of epidermal growth factor receptor (erlotinib, gefitinib, and afatinib), ALK-cMET (crizotinib), PDGFR/BCR-Abl (dasatinib), and multikinase inhibitors (sunitinib and sorafenib). In clinical samples, we measured the disposition of each compound within various blood compartments. *Methods:* we used an optimized CaCo2 gut epithelial model to characterize 20 µM TKI absorption. The apical/basolateral transfer is considered to represent the gut/blood transfer. Drugs were measured using LC-MS/MS. *Results:* sorafenib and sunitinib showed the highest apical/basolateral transfer (P*app* 14.1 and 7.7 × 10^−6^ cm/s, respectively), followed by dasatinib (3.4), afatinib (1.5), gefitinib (0.38), erlotinib (0.13), and crizotinib (n.d.). However, the net absorptions for dasatinib, afatinib, crizotinib, and erlotinib were highly negative (efflux ratios >5) or neutral/negative, sorafenib (0.86), gefitinib (1.0), and sunitinib (1.6). A high negative absorption may result in resistance because of a poor exposure of tissues to the drug. Accumulation of the TKIs at the end of the transfer period (A->B) was not detectable for erlotinib, very low for afatinib 0.45 pmol/μg protein), followed by gefitinib (0.79), dasatinib (1.1), sorafenib (1.65), and crizotinib (2.11), being highest for sunitinib (11.9). A similar pattern was found for accumulation of these drugs in other colon cell lines, WiDr and HT29. In clinical samples, drugs accumulated consistently in red blood cells; blood to plasma ratios were all >3 (sorafenib) or over 30 for erlotinib. *Conclusions:* TKIs are consistently poorly absorbed, but accumulation in red blood cells seems to compensate for this.

## 1. Introduction

Tyrosine kinase inhibitors (TKIs) are a relatively new group of compounds being used for the treatment of various forms of cancer. TKIs typically are small molecules derived from a 4-anilinoquinazoline core structure, ranging from 250 to 600 amu [1]. TKIs are marketed as being more tumor specific than traditional systemic chemotherapy and are often referred to as “targeted therapy.” Therefore, TKIs require highly specific genetic properties in order to be effective [1]. This limits their general use to only approximately 3–4% of the target population group and many patients need to be genetically screened to select the most effective drug for each patient. In addition, TKIs also suffer from poor physical chemistry properties such as poor solubility, since all TKIs are virtually insoluble in water [2]. This presents problems with administration since they are administered orally on an out-patient basis. Only limited preclinical and even less clinical data are available on the bioavailability of each individual TKI across the gut membrane. This is due to difficulties with solubility limiting IV preparations (Table 1), which preclude the use of a similar dose for IV and oral administration that would be the proper procedure. Most bioavailability data (when available) were determined by extrapolation (e.g., different doses at IV and oral). Sometimes reaching appropriate plasma concentration (meaning sufficient to inhibit the target) was defined as good bioavailability. 

Investigations into the absorption of compounds into systemic circulation is a well-documented field, but absorption is subject to many variables [33,34,35,36,37]. However, for all forms of chemotherapy, it holds that the drug has to reach the tumor and its target. This may not only be due to a limited uptake in the target tissue, but its tendency to be effluxed, e.g., by one of the ATP binding cassette (ABC)-efflux transporters, either by the tumor or back into the gut may be much more important [37]. A poor uptake from the gut may lead to resistance. 

**Figure 1:**
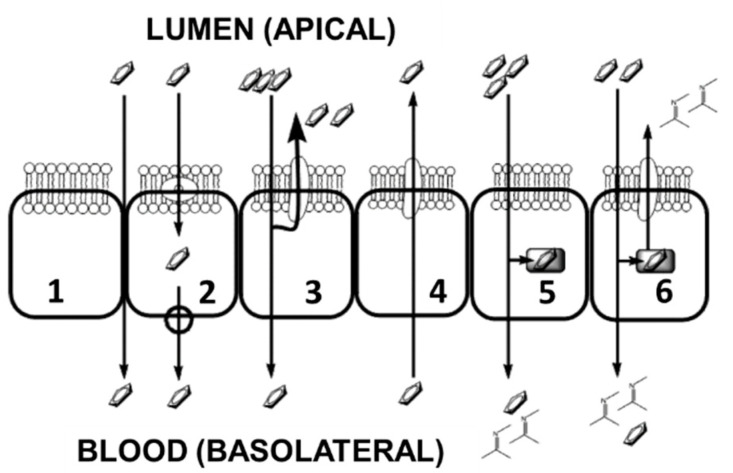
Graphical representation of the six epithelial transport characteristics for the uptake of cytotoxic compounds from oral administration, adapted from Chan et al. [37]. 1—Paracellular absorption via the interstitial spaces between cells, this is restricted by intercellular tight junctions. 2—Transport mechanisms at either the apical or the basolateral membranes. 3—Restriction of transport into the blood flow by apical membrane efflux transporters. 4—Efflux membrane transporters enabling clearance of compounds from the blood. 5—Metabolism of compounds prior to transport to the blood. 6—Co-ordination of metabolizing enzymes and apical efflux transporters forming an intestinal absorption barrier.

TKIs are all marketed as oral administration (with or without food intake being compound specific) making them noninvasive, convenient, and theoretically more cost effective. TKI bioavailability is limited by the absorption process depending on environmental conditions, specific membrane transport systems, and intracellular metabolizing enzymes. The key variability to TKI systemic circulatory levels lies within the absorption characteristics of the small intestine which are dependent on the physicochemical properties of each individual molecule [2]. The intestinal epithelium is a highly effective barrier to the entry of compounds into the blood with basic functions being separated into six (but not limited to) different transcellular and paracellular processes (Figure 1). Epithelial cells are polarized into an apical membrane (exposed to the gut contents) and a basolateral membrane (access to the circulating blood flow), with differing transport proteins. The epithelium cellular structure contains tight junctions limiting drug entry via the paracellular process (entry via the gaps between cells by diffusion). The transcellular process involves the uptake across the apical membrane followed by transport across the cytosol and finally movement back out of the cell across the basolateral membrane into the blood flow. However, once a compound has crossed into the cytosol, several processes may limit its efficacy, since it becomes exposed (1) to apical efflux ABC transporters, which move the compound back into the gut; (2) to metabolizing enzymes, which could change the compound to a more suitable form for the apical transporters; (3) to metabolizing enzymes that deactivate the compound’s chemotherapeutic effect; (4) to sequestration into internal organelles or vesicles; (5) to membrane efflux ABC transporters, which can mediate direct blood to gut secretion.

To combat these problems and to try to gain an insight into the variability of clinical absorption, we adapted existing methodology using an optimized in vitro Transwell system to investigate the transport across a CaCo2 monolayer (representing the gut epithelial system) [38,39]. The major difference is the shortening of the establishment of the Transwell from 20 to 3 days, as described earlier [38]. This Transwell system performed similarly to the systems described in the literature and used by various pharmaceutical companies, who consider CaCo2 as the standard model for studying gut epithelial transfer. This Transwell system acts as a model system for gut transport and was used to test the uptake characteristics of gefitinib and erlotinib [39]. Although the system has important advantages, there are several limitations such as variable expression of several transporters, differences in morphology compared to normal gut epithelial, and variable paracellular transfer. However, since the system is widely used, it is considered as one of the best representative models. The inner well represents the gut compartment and is separated from the outer well, representing the blood compartment, by the CaCo2 monolayer. The wells contain medium + drug or just medium (Figure 1 and Figure S1). The bipolar nature of the CaCo2 cell line permits the investigation of the transport characteristics from the theoretical gut to the epithelial lining and from the epithelial lining back into the gut compartment.

**Figure 2:**
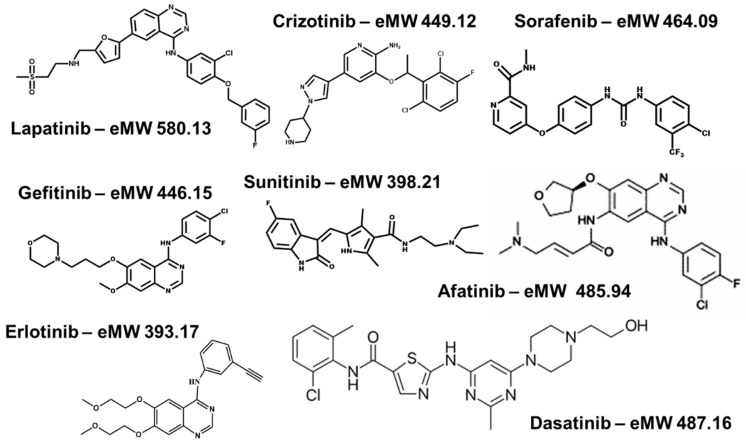
Molecular structures of the compounds under investigation for transport characteristics.

From seven FDA approved compounds (gefitinib, erlotinib, sorafenib, sunitinib, dasatinib, afatinib, and crizotinib; Figure 2 and Table 1), we determined perfusion coefficients using the Transwell system. Gefitinib and erlotinib are first-generation reversible small molecule inhibitors of the epidermal growth factor receptor (EGFR) tyrosine kinase. Afatinib is a second-generation small-molecule inhibitor also targeting EGFR [40]. The drugs are registered for the treatment of adenocarcinoma of non-small cell lung cancer (NSCLC) with activating EGFR mutations. Sorafenib and sunitinib are orally active multikinase inhibitors approved for the treatment of hepatocellular carcinoma and renal cell carcinoma, respectively [41,42]. Crizotinib is an echinoderm microtubule-associated protein-like 4 (EML4)-anaplastic lymphoma kinase (ALK) and cMET inhibitor that has been approved for use in NSCLC [43], while dasatinib is a platelet-derived growth factor receptor/breakpoint cluster region-Abelson murine leukemia oncogene homolog-1; (PDGFR/BCR-Abl) inhibitor registered for use in chronic myeloid leukemia in a selected population [44]. Physicochemical properties of these compounds determine whether they are good substrates for various influx- and efflux transporters, responsible for intestinal uptake, or whether they may compartmentalize in cellular organelles leading to resistance [45].

Using the derived perfusion coefficients (P*app*) and associated efflux ratios, we aimed to explain characteristics of the clinically observed pharmacokinetic properties possibly responsible for resistance. For this purpose, we also analyzed, in a limited number of patients, the concentrations of several of these drugs in both plasma and a neglected blood compartment, the red blood cells. Not much is known about drug transport and delivery of TKIs in red blood cells despite evidence suggesting a crucial role for this compartment in the pharmacokinetics for several TKIs [19,46]. 

## 2. Results

### 2.1. EGFR Inhibitors: Gefitinib, Erlotinib, and Afatinib: Apical to Basolateral and the Reverse

**Figure 3:**
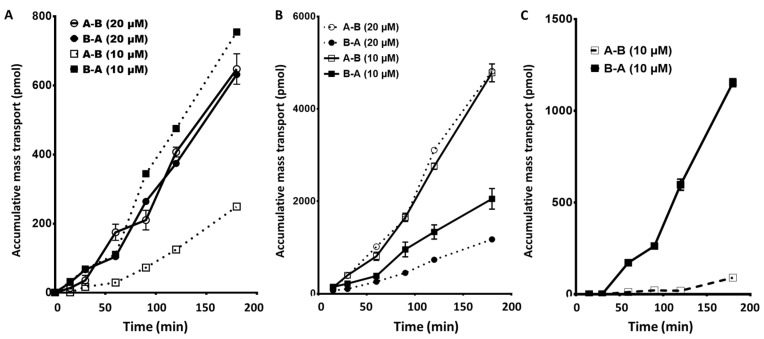
Cumulative transport of gefitinib (**A**), erlotinib (**B**), and afatinib (**C**) across a CaCo2 monolayer at both 10 and 20 µM concentration in the apical to basolateral (A–B) and the basolateral to apical (B–A) directions. Values are the mean ± standard error of the mean of 3 replicates. When not visible, the error bars are within the size of the marker.

Transport of gefitinib (10 µM) in the apical to basolateral direction (Figure 3A) showed a clear time-dependent linear increase in drug across the membrane. The accumulative mass transported represents a perfusion coefficient (P*app*) of 0.57 µm/s in the uptake direction. This is somewhat higher compared to the previously reported 20 µM perfusion coefficient of 0.38 µm/s but was not significantly different [39]. However, the total amount accumulation over the 3 h period was approximately 50% of the previously reported 20 µM perfusion experiment. This indicated a dose-related absorption effect in the apical to basolateral direction, possibly because at higher concentrations, gefitinib inhibited its passage through the cell towards the basolateral side suggesting a combination of active and passive transport mechanisms.

Transport of 10 µM erlotinib (Figure 3B) showed a linear time-dependent increase, which was comparable to previously reported 20 µM perfusion experiments [39], but the total amount transported at the 10 µM dosing level was approximately 50% compared to previous values. This indicated a concentration-dependent transport mechanism consistent with the conclusion that erlotinib uptake is predominately passive diffusion. The perfusion rate at the 10 µM level was higher compared to the 20 µM level (apical to basolateral P*app* (10 µM) of 0.48 µm/s compared to P*app* (20 µM) of 0.13 µm/s). However, for the three experiments performed, no significant difference was determined between the 10 and 20 µM transfer rates (*p* = 0.13). 

Afatinib was only tested at the 10 µM level demonstrating a perfusion rate at lower levels compared to gefitinib (P*app* of 1.48 µm/s, 90 pmol accumulated in 3 h). However, the observed transfer showed a consistent decrease during the 1–2 h period before increasing again over the last hour. 

The transport characteristics of 10 µM gefitinib in the reverse direction (Basolateral to Apical; B–A, Figure 3A) demonstrated a perfusion coefficient (P*app*) of 0.19 µm/s. Therefore, the net efflux ratio of gefitinib at a dose level of 10 µM was 0.33. However, at 20 µM, the P*app* for gefitinib was similar in both directions (flux ratio 1.0), whereas at the 10 µM concentration, the flux ratio shows a net influx of gefitinib. This suggests that transport of gefitinib is directly proportional to the concentration in the gut and transport characteristics change at different concentration levels. Variations in localized concentrations of gefitinib at the boundary layer of the gut epithelial cells may affect absorption variability.

Previously reported data indicated that for erlotinib at 20 µM, the B–A transport was significantly greater in comparison to A–B (P*app* of 0.13 µm/s vs. P*app* of 0.70 µm/ s, *p* < 0.009) with an efflux ratio of 5.6, indicating a very strong apical flux flow. However, at the lower 10 µM, the A–B influx of erlotinib was increased, whereas the B–A efflux remained relatively the same, so that efflux ratio decreased to 1.6. This demonstrates a net gain in systemic absorption at the lower concentration of drug, which is not consistent with a purely passive diffusion uptake (Figure 3B).

Similar to the results observed for erlotinib, afatinib demonstrated a significantly higher B–A perfusion rate (P*app* of 10.9 µm/s, *p* = 0.039) compared to the A–B rate (P*app* of 1.5 µm/s), resulting in an efflux ratio of 7.34 (Figure 3C). 

### 2.2. Sunitinib, a Vascular Endothelial Growth Factor Receptor (VEGFR) Inhibitor, and Sorafenib, a Platelet-Derived Growth Factor Receptor (PDGFR): Apical to Basolateral and the Reverse

Both sunitinib and sorafenib (7.7 and 14.1 µm/s) demonstrated a greater gut to systemic circulation influx compared to gefitinib and erlotinib (range of 20–110-fold higher). Sunitinib (20 µM) demonstrated a linear transfer in the apical to basolateral (r2 = 0.991) reaching 581.2 pmol transferred in the 3-h period with a P*app* (20 µM) of 7.7 µm/s. Sorafenib did not show a completely linear transfer relationship, demonstrating an increasing transfer over the time period measured. Total amount transferred in the 3-h period was approximately 950 pmol, which was a third higher than sunitinib. 

**Figure 4:**
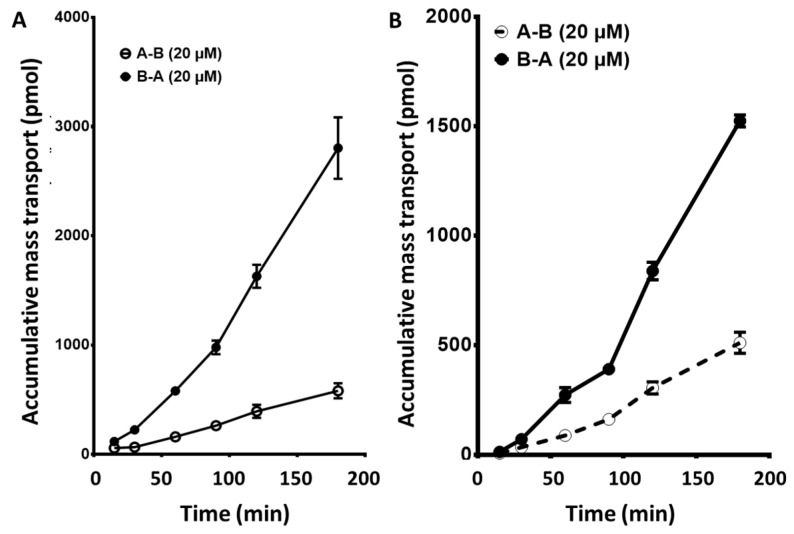
Cumulative mass transport of sunitinib (**A**) and sorafenib (**B**) across a CaCo2 monolayer. Values are the mean ± standard error of the mean of 3 or more replicates. When not visible, the error bars are within the size of the marker.

Transfer of sorafenib and sunitinib was not linear in the basolateral to apical direction, demonstrating a progressively increasing rate of transfer over the 3-h period for both drugs. The total amount of drug transferred was significantly more than in the apical to basolateral direction, however, the average efflux ratios were 0.8 and 1.6, respectively (Figure 4A,B).

### 2.3. Crizotinib, an EML4-ALK and c-MET Inhibitor, and Dasatinib, a PDGFR and a BCR-Abl Inhibitor: Apical to Basolateral and the Reverse

**Figure 5:**
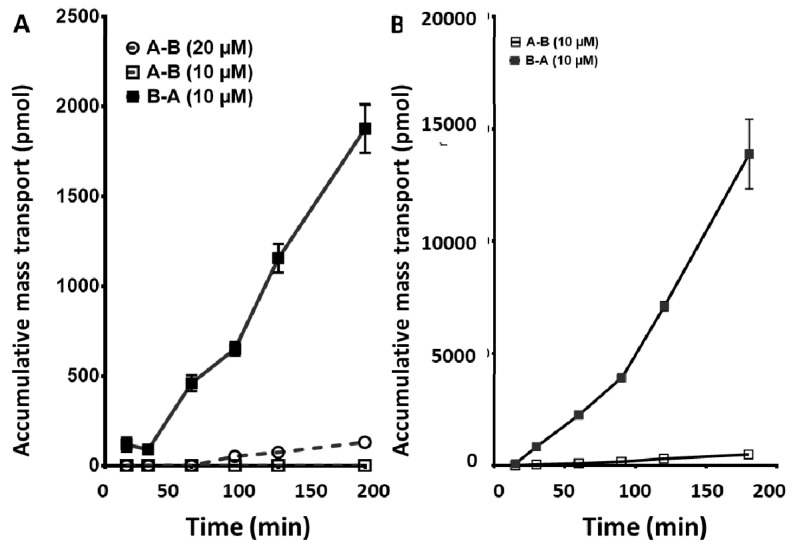
Cumulative mass transport of crizotinib (**A**) and dasatinib (**B**) across a CaCo2 monolayer. Crizotinib was tested at 10 and 20 µM. At 10 µM, crizotinib no A–B transport was detected, most likely due to the sensitivity of the assay used for determination of the concentration. At a higher concentration (20 µM), minimal A–B transport was observed but was still 20-fold lower than mass transport observed in the B–A direction at the 10 µM dose level. Dasatinib was tested at 10 µM. All values are the mean ± standard error of the mean 3 or more replicates. When not visible, the error bars are within the size of the marker.

Since 20 µM crizotinib exposure caused cell death and disruption of the membrane, the concentration of crizotinib was reduced to 10 µM. However, for the apical to basolateral samples, nothing could be detected. This was not considered as zero transfer (or uptake) but an analytical sensitivity problem with crizotinib. Under the detection parameters used, sensitivity was too low compared to all the other compounds and the levels transferred too low in the time permitted to be seen. Conversely, in the basolateral to apical direction the accumulated amount of crizotinib was high and the transfer seen was linear in nature (Figure 5A). 

Transport characteristics for dasatinib in the direction of apical to basolateral was the lowest of the seven compounds measured (except for the undetectable crizotinib) with an average total accumulation of 499.3 pmol, in the 3-h transfer period, and P*app* (20 µM) of 3.4 µm/s. Transfer conformed to a second-order polynomial rate with an average of 3.4 µm/s (Figure 5B).

In the reverse situation (basolateral to apical), significantly more dasatinib was transferred (P*app* (20 µM) of 20.4 µm/s) across the monolayer. This equated to an average total accumulation of 13,889 pmol over a 3-h period, this was significantly different to the A–B observed transfer rates (*p* < 0.006, one tailed paired *t*-test), and the efflux ratio was 6.0.

### 2.4. Intracellular Accumulation of TKIs in the CaCo2 Transwell Cells and in Various Colon Cancer Cell Lines

**Figure 6:**
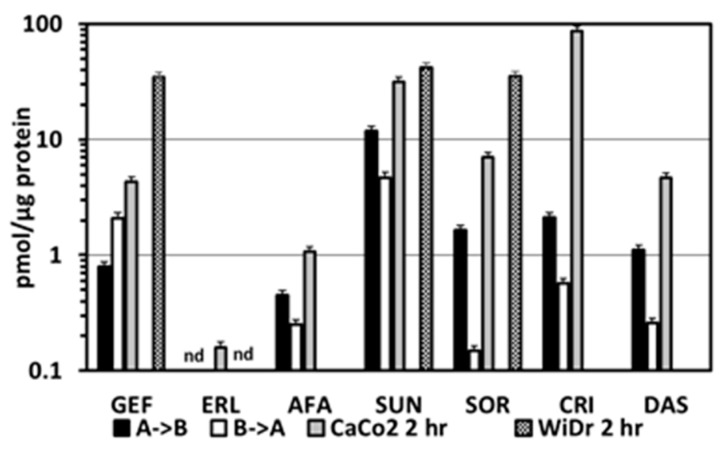
Accumulation of the TKIs in CaCo2 cells harvested at the end of the mass transport experiments in comparison to TKI accumulation in CaCo2 cells and WiDr colon cancer both grown as normal monolayers. These cells were exposed to 20 μM of a TKI and were harvested after 2 h for determination of TKI concentration with LC-MS-MS. Values are means ± SEM for the number of experiments described in Figure 3, Figure 4, and Figure 5 and from three separate experiments for the monolayer CaCo2 and WiDr cells. GEF, gefitinib; ERL, erlotinib; AFA, afatinib; SUN, sunitinib; SOR, sorafenib; CRI, crizotinib; DAS, dasatinib; nd, not detectable. A->B, mass transport from A to B; B->A, mass transport from B to A. For AFA, CRI, and DAS, no data in WiDr cells were available.

In order to get insight in the barrier role of the CaCo2 model system, we determined the intracellular accumulation of the TKISs at the end of the transport period, in normal monolayers of CaCo2 cells and in several other colon cancer cell lines. At the end of the transport period (both from the B->A and A->B incubations), cells were harvested and intracellular accumulation was determined. These data were compared to accumulation in CaCo2 cells grown as normal monolayers (Figure 6). In line with the higher mass transport, the intracellular accumulation for A->B was higher for afatinib, sunitinib, sorafenib, crizotinib, and dasatinib than for B->A. For gefitinib, the accumulation was higher for the B->A experiments, while no erlotinib was detectable for both directions. The highest accumulation was observed for sunitinib. When CaCo2 cells were incubated as monolayers and exposed to a similar drug concentration, the same pattern was found as for the A->B incubations. Although sunitinib was one of the highest, the accumulation of crizotinib was higher, while that of erlotinib was just detectable. In WiDr cells, a similar pattern was found as in CaCo2 cells, with no erlotinib and with sunitinib being highest, followed by sorafenib. In another colon cancer cell line, HT29 accumulation after 24 h showed a similar accumulation pattern with very low erlotinib ((0.035 pmol/μg protein), a low dasatinib (0.14 pmol/μg protein), intermediate sorafenib (6 pmol/μg protein), and high sunitinib (32.2 pmol/μg protein).

### 2.5. Blood Compartments

Since standard blood sampling for pharmacokinetics is usually limited to plasma samples, we were only able to obtain other blood compartments from a limited number of selected patients depending on the TKI. In general, plasma concentrations reached their steady state level relatively fast, which is in line with the rate of passage that we is seen for most drugs. Large interindividual differences were found, which may be related to, e.g., food intake, ethnic and regional differences [19,30], comedication (not always defined), different transporter expression (which may be affected by comedication), and protein binding, as defined earlier [2] (Table 1). 

**Figure 7:**
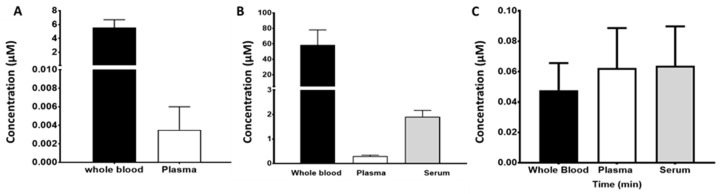
The steady state concentrations of gefitinib (**A**), erlotinib (**B**), and afatinib (**C**) in different blood compartments. Samples for gefitinib (whole blood and plasma) were obtained after an average of 124 ± 67 days at 250 mg per dose level. Samples for erlotinib were obtained after an average of 238 ± 167 days at 150 mg per dose level. Samples for afatinib were obtained after 4 days following standard 40 mg oral dosing per day. Values are the means ± standard error of the mean of 4 or more replicates as indicated in the text.

In six patients with non-small cell lung cancer (NSCLC) treated with gefitinib, corresponding plasma and whole blood samples were taken at a single time point during steady state treatment (124.4 ± 67 days). Concentrations in the whole blood samples showed a markedly higher level compared to the plasma, possibly because of the high red blood cell accumulation (Figure 7A), indicating that the red blood cell to plasma ratio plays a very important role in the variability of gefitinib pharmacokinetics possibly leading to variable efficacy of the drug.

In 32 NSCLC patients treated with erlotinib, steady-state concentrations of plasma and serum were determined as well as whole blood for five patients (Figure 7B). The concentrations of erlotinib in plasma were significantly (*p* < 0.05) lower compared to that in whole blood and serum. The data suggest that red blood cell uptake of erlotinib is a variable not considered in all the published pharmacokinetic studies and could be a key factor behind inter- or intrapatient variability. 

In four patients with NSCLC, steady state concentrations of afatinib were determined in whole blood, plasma, and serum. Variation in steady state concentrations between the four patients was quite large but the resulting average values indicate that afatinib is not accumulated within the red blood cell compartment to the same degree as gefitinib and erlotinib (Figure 7C).

**Figure 8:**
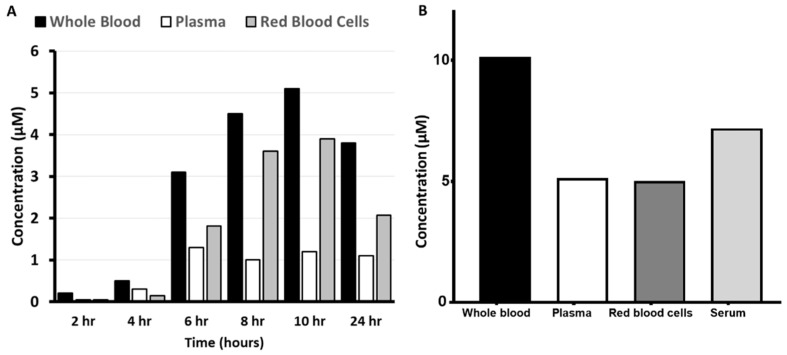
Plasma pharmacokinetics of sunitinib (**A**) and sorafenib (**B**). Samples for sunitinib were obtained over the first 24 h after drug ingestion from one single naïve patient with an advanced solid tumor, refractory to standard treatment, receiving oral sunitinib at 700 mg per week. Samples for sorafenib were obtained at steady state conditions (30 days after initiation of treatment) from one representative patient receiving 400 mg sorafenib twice daily.

Sunitinib was determined in plasma, serum, and whole blood after a standard dosing regimen; plasma and serum showed comparable levels, while whole blood was 40–50% lower than plasma. This suggests that sunitinib does not accumulate within the red blood cells under the conditions tested. However, in an additional cohort of 7 patients, a single sunitinib dose, 14-fold higher than standard, was investigated for pharmacokinetic properties. Data from one representative patient showed a clear accumulation in red blood cells (Figure 8A). In the total cohort of 7 patients, the whole blood/plasma ratio was 2.6 ± 0.7 (mean ± SD) as compared to 0.7 for standard treatment at 50 mg. Sunitinib accumulation could also be verified visually since red blood cells changed their color (yellowish) because of this intrinsic property of sunitinib, which was not feasible with the other drugs. 

Sorafenib demonstrated a 2–4-fold increase in the concentration of the drug in the whole blood compared to the plasma, which was observed in several patients at this low dose (Figure 8B). However, at higher doses (2000–2800 mg cumulative daily dose), the whole blood/plasma ratio decreased to 1.2 ± 0.4 (mean ± SD of 12 patients). There was no difference in the ratio between the three cycles given to these patients. 

**Figure 9:**
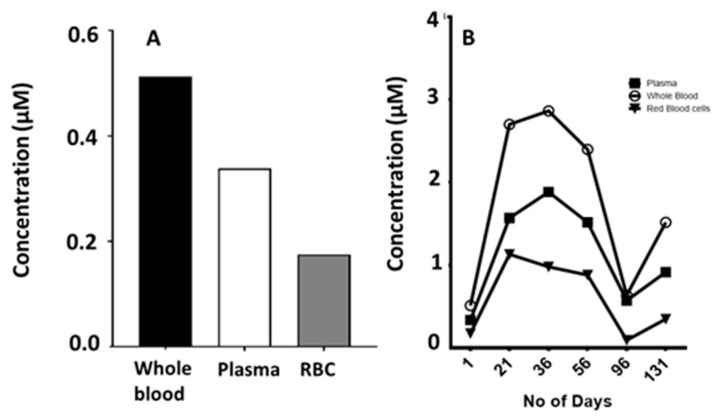
Crizotinib disposition in the blood components following standard dosing (250 mg twice daily) of a single NSCLC patient at the start (day 1) of treatment (**A**). Blood compartment disposition in time after 1, 2, 3, and 6 months of crizotinib treatment (**B**).

The disposition of crizotinib within the blood compartment following a standard dose demonstrated concentrations in whole blood 30% higher than in the corresponding plasma, indicating an accumulation within the red blood cell compartment. Analysis of similar samples in the same patient over a 6-month period revealed a changing blood to plasma ratio (Figure 9B).

## 3. Discussion

In this paper, we demonstrate a large variation in the gut epithelial transport of 7 different TKIs as determined with the CaCo2 model system (Table 2). For various drugs, the basolateral-apical transport (comparable to transport to the gut) was higher than apical–basolateral transfer from the gut. Earlier, we reported a large variation in the effect of drug efflux inhibitors, which demonstrated that transport is neither all passive diffusion or limited to a single transport mechanism [38,39]. However, it can be suggested that for gefitinib with the decreased efflux ratio (0.46), after the treatment with NaN3, several active ATP transport mechanisms on both the apical and basolateral membranes are essential in the transfer of gefitinib across the cellular membranes. This is also observable to differing degrees for erlotinib, afatinib, sorafenib, dasatinib, sunitinib, and crizotinib. Both afatinib and dasatinib demonstrated extreme differences in the transfer of the drug across the CaCo2 membrane with B–A transport >6-fold higher than the A–B transport. This phenomenon is reflected in the pharmacokinetic plasma levels seen in the clinic (Table 1) with erlotinib and sorafenib demonstrating higher plasma levels compared to afatinib and dasatinib with 10-fold lower levels. Extending this further with reference to the tumor, these results suggest that dasatinib would not accumulate sufficiently within solid tumor cells to any significant degree possibly explaining the inefficacy of, e.g., dasatinib in solid tumors [47,48]. Since dasatinib can inhibit cell growth at submicromolar concentrations (with 60 nM leading to 50% growth inhibition after 72 hr exposure in WiDr cells) the plasma concentrations of dasatinib are high enough to exert an antileukemic effect. In line with these data, dasatinib also showed a very low accumulation not only in the CaCo2 cells at the end of the experiment but also in HT29 cells. The concentrations of various TKIs in several cell lines after a 2- or 24 h incubation also showed a very low accumulation of erlotinib for every cell line, a somewhat higher accumulation of afatinib but a very high accumulation of crizotinib and sunitinib (Figure 5). Afatinib and erlotinib show a fair degree of clinical efficacy, which can possibly be explained by the location of the target receptor for these drugs at the membrane (EGFR). The high accumulation of crizotinib and sunitinib is related to the phenomenon of lysosomal accumulation [2,45], which was also observed in other types of cells, such as NSCLC [49], breast cancer, and gliomas (Figure S2).

The average P*app* for both gefitinib and erlotinib in the apical to basolateral direction at the 10 µM concentration was greater than at the 20 µM concentration, indicating a mechanism that is blocked at higher concentrations, whereas in the basolateral to apical direction, the transfer rate for gefitinib decreased at the lower concentration indicating a better retention of the drug for systemic circulation. The similarity of the amounts transferred in the 3 h time period when comparing two concentrations indicates a mechanism that is easily overloaded. For erlotinib, the effect was much less apparent indicating a less important role for active transport mechanisms although some evidence suggests that transport is not solely passive. This difference in transfer at various concentrations reveals a complicated series of mechanisms, which are directly related to the concentration of drug at the interface of the gut epithelial wall, e.g., erlotinib hardly accumulated in the CaCo2 cells at the end of the experiment (Figure 6), despite a proper transfer. This indicates that erlotinib’s transfer is partially through the cells but apparently to a much larger extent “between” the cells indicating a paracellular transport. 

Considering that most TKIs are dosed in the fasting state, the tablet dissolution and the mixing of the released drug with the gut contents is a crucial aspect of the drug absorption. Different conditions in the gut just after dosing will significantly affect the absorption rate [50], which will subsequently affect the circulation concentration of the drugs. These variables are uncontrollable in the clinical situation and will lead to variable systemic concentrations. Moreover, it is now well known that the gut microbiome may affect drug metabolism and disposition, sometimes leading to drug resistance [51]. A poor absorption will obviously lead to a low systemic exposure and resistance to treatment. Although administration of TKIs is usually recommended under fasting conditions there is accumulating evidence that the addition of food will increase uptake in most cases (Table 1), while the type of food intake (i.e., moderate European) is important [14,27,30,50,52]. We previously demonstrated that the presence of protein in the media for translational investigations is important to achieve reproducible transport concentrations [39]. Although most TKIs are given at low daily doses, administration at a high weekly dose may give a better pharmacokinetic profile, leading to a higher tissue accumulation, bypassing resistance. This will depend on the physicochemical properties (Table 2), since for sunitinib, a high-dose pulsatile dosing enabled to increase the dose considerably leading to an antitumor effect in tumors considered to be resistant to this drug [53]. However, for sorafenib, which has different properties (Table 2 [45]), a high dose was not tolerable [54].

In addition to the absorption variability suggested by the membrane investigation, another peculiarity was observed with these compounds. Although for some drugs we were only able to study more blood compartments in a limited number of patients, it was clear that for some of the compounds, the concentration in the whole blood was significantly higher (*p* < 0.0001) than that found in the plasma. Equally curious was the fact that serum concentrations were also greater compared to plasma but less than that found in the whole blood. This indicated that serum is not a good matrix for the pharmacokinetic analysis of these drugs. At the standard dosing regimens, it was observed in clinical samples that erlotinib was 100-fold higher in the whole blood compared to plasma. It can be assumed that the difference in concentration between the two matrixes must in part be due to the uptake of the compound in the red blood cells. Similar to erlotinib, both gefitinib and sorafenib showed a higher red blood cell uptake although not to the same degree as for erlotinib. For some of the TKIs, the number of samples available for analysis of matched plasma, serum, and whole blood samples was limited. However, the extended data sets for some drugs support earlier findings for several drugs (including anticancer drugs) that the red blood cell content is extremely important in the pharmacokinetic analysis [55] and may even explain why several studies have shown no correlation between plasma peak levels and drug efficacy. 

The CaCo2 gut epithelial system for the investigation of gut transfer properties provides an excellent translational system for TKIs, as was earlier shown for a large variety of compounds, in which it was shown that the CaCo2 model provides an excellent model system to study gut epithelial drug transfer [56]. We have simplified the widely used CaCo2 model, enabling to reduce culture time from 20 to 3 days, with similar properties as the 20-day model [38,39]. The data reveals complex transport mechanisms, which vary between each compound. Indications of the involvement of both passive and active mechanisms [38] operating on concentration-related parameters complicate the characterization of the exact mechanism but goes a long way towards explaining why these compounds can show highly variable absorption characteristics within the clinical population. The changing blood to plasma ratio of drugs demonstrates that red blood cells play an important role in the transport of these compounds around the body, while also demonstrating another variable factor in the pharmacokinetic analysis not taken into account for many of the published pharmacokinetic profiles.

It can be concluded that the variable nature of the uptake for TKIs is due to a number of factors that have been neglected during investigations and could account for the contradictory pharmacokinetic results and varying degrees of resistance that have been observed in different subsets of the cancer populations. It is recommended for future pharmacokinetic studies that all blood compartments are measured especially considering the growing popularity of dry blood spot testing for pharmacokinetics.

## 4. Materials and Methods 

### 4.1. Materials, Equipment and Analysis

High-purity dasatinib, crizotinib, afatinib, sunitinib, sorafenib, erlotinib, and gefitinib were purchased from LC Laboratories (Woburn, MA, USA). All stock solutions were prepared in dimethyl sulfoxide (DMSO), and subsequently, standard dilutions were made in 100% ethanol; all dilutions were stored at −20 °C. Ko143 was a kind gift of Professor G.J. Koomen, University of Amsterdam, The Netherlands. All other reagents such as fetal bovine serum and the various different growth/transfer mediums were purchased as reported previously for the description of the model system [39]. Analytical grade solvents such as DMSO, ethanol, acetonitrile, methanol, and isopropanol were purchased from Biosolve BV (Valkenswaard, The Netherlands). 

The BioCoat^®^ HTS Caco-2 Assay System and BD Falcon^TM^ 24-well Multiwell Plates were purchased from Becton Dickinson BV (Breda, The Netherlands), while the Trans Epithelial Electrical Resistance (TEER) meter (Millicell^®^-ERS) was provided by Millipore (Amsterdam, The Netherlands). 

Analyses were performed using a Dionex Ultimate 3000 chromatographic system (Fischer Scientific, Breda, The Netherlands) coupled with an API 3000 mass spectrometer (AB Sciex, Nieuwerkerk ad IJssel, The Netherlands) in combination with Analyst software (version 1.6.3, Dionex, Sunnyville, CA, USA) and Chromeleon LC modules version 6.8, controlled by Dionex Mass link (DMS) version 2.10 [57].

Protein precipitation with acetonitrile (1:5) was performed for each sample and standard preparation as reported previously [57]. 

Liquid chromatography coupled to tandem mass spectrometry (LC-MS/MS) analysis was performed with a mobile phase consisting of acetonitrile, ammonium acetate (20 mM, pH 7.8), and methanol in the ratio of 66.1:24.5:8.3% (*v/v*) with 1% isopropyl alcohol added as a chromatographic modifier. Chromatographic separation was obtained with a Phenomenex prodigy ODS3 column, 3 µm particle sizes, 100 × 2.00 mm (Phenomenex, Utrecht, The Netherlands) at a flow rate of 0.25 mL/min [57].

### 4.2. Cell Culture Models and the CaCo2 Transwell Model

The Transwell system was developed using the wild type Caco-2 cell line (passage 15–25) cultured in Dulbecco’s Minimal essential medium (DMEM) at standard conditions of 37 °C, 5% CO_2_, and 100% humidity as previously reported [39]. Prior to transfer studies, cells were seeded on a BioCoat 24 well Transwell plate and incubated for 20–24 h with growth medium and then for 44–48 h with differentiation medium. To ensure a controlled growth environment the plate was covered with Breathe-Easier cell culture foil during the incubation period. The integrity of the developed monolayer was assessed by TEER before administration of compound solutions and after the final sampling stage.

Transport studies of 10 µM gefitinib, erlotinib, afatinib, sunitinib, sorafenib, crizotinib, and dasatinib were performed in the direction apical to basolateral (A–B) and from basolateral to apical (B–A). The initial concentration of the drug was verified from a 20 µL sample taken immediately from the donor compartment after drug administration (*t* = 0). Subsequently, samples (50 µL) were taken from each receiver compartment at the time points 15, 30, 60, 90, 120, and 180 min after drug administration. A final sample of 20 µL was taken from the donor compartment after 180 min.

In a different set of experiments, the CaCo2 cells were cultured in 6-wells plates as monolayers until semiconfluency [58]. Two other colon cancer cell lines, WiDr and HT29, were cultured similarly. These cells were exposed for 2 h to various TKIs at concentrations varying from 50 nM to 20 µM). Since the TKIs showed a linear uptake for this short time period and were not toxic to the cells in this period, we decided to report accumulation data at 10 µM. At the end of the incubation, cells were harvested, as described earlier [38], and extracted for drug analysis using LC-MS-MS as described above. 

### 4.3. Calculations and Statistics

The permeability coefficient (P*app*) represents a measure for the efficiency of transport and was calculated with the following equation:

$$Papp=\frac{\Delta Q}{\Delta t}\times \frac{V}{C_{0}\cdot A}$$

where ∆Q/∆t = the rate of increase in drug concentration (accumulative mass transport) in the receiver compartment over time (µM/s), V = volume in the receiver compartment (mL), C_0_ = the initial concentration of drug in the donor compartment (µM), and A = the membrane surface area (cm^2^).

To take into account the slightly nonlinear transfer characteristics of each compound, the rate of increase in drug concentration was determined for each time period and the P*app* was then calculated. The average P*app* was then determined with units of cm/s × 10^−6^ or µm/s.

The efflux ratio was calculated according to the equation:

$$\mathrm{Efflux}\;\mathrm{ratio}=\frac{PappB\to A}{PappA\to B}.$$

Efflux ratios > 1 indicate a higher drug flux from B to A. The P*app* ratios are shown as a mean value of three or more measurements ± SEM. Statistical significance was determined using a simple Student’s *t*-test where a *p* value of <0.05 was considered significant.

### 4.4. Blood Pharmacokinetics

For analysis of matching whole blood and separated plasma samples, we were able to obtain whole blood, plasma, and serum from several patients for most of the TKIs, since standard blood sampling was limited to plasma. Analysis was performed for patients treated with either gefitinib, erlotinib, sorafenib, sunitinib, afatinib, or crizotinib. For gefitinib, 5 Caucasian adults with untreated advanced NSCLC were treated with daily administration of 250 mg gefitinib or 150 mg of erlotinib. A single blood sample was taken after an average of 124 ± 67 days (gefitinib) or 238 ± 167 days (erlotinib). Each individual sample was divided into two components; a 1 mL of tube of whole blood and 0.5 of plasma, each sample was stored at −20 °C until analysis could be performed. An additional serum sample was also obtained for patients treated with erlotinib [59]. Blood samples for afatinib were obtained from NSCLC patients after 4 days receiving the standard daily dose of 40 mg.

For sorafenib, a single male patient with metastatic renal cell carcinoma was initially treated with 50 mg per day sunitinib but due to toxicity issues this dose was reduced to 25 mg and eventually treatment halted [60]. Subsequent treatment was with 200 mg sorafenib per day, which was escalated to 400 mg twice per day after no toxicity issues were observed. Blood samples were taken at 1, 2, and 30 days after the start of treatment. Samples were separated from the whole blood into plasma, serum, and a packed red blood cell pellet (using the MESED apparatus [61]). In a dose escalation study in order to determine whether a high pulsatile dose of sorafenib could be given to patients, we analyzed red blood cells and whole blood in 12 patients treated with sorafenib at total doses ranging from 2000 to 2800 mg daily [54]. From these patients, samples were collected in 3 cycles.

For sunitinib, initially pharmacokinetic samples were collected over the first 24 h period from a single male patient, with a renal cell carcinoma receiving a nonstandard treatment schedule (700 mg biweekly) [53], in comparison to the standard 50 mg daily dose. Whole blood samples were divided into plasma, serum, and a packed red blood cell pellet. This cohort could later be extended with 6 patients treated with 800 mg sunitinib every 2 weeks enrolled in a clinical study to determine the tolerability of pulsatile sunitinib.

The difference in crizotinib blood compartmentalization was estimated in a single Caucasian patient with locally advanced NSCLC that was anaplastic lymphoma kinase positive following treatment with the standard 250 mg twice daily. Five blood samples were taken over a 5-month period and divided into whole blood, an extracted 100 µL of red blood cells, and a plasma sample. 

## 5. Conclusions

Most TKIs have a poor oral bioavailability. We reasoned that this was due to poor intestinal absorption, which was investigated using an optimized CaCo2 Transwell system. With this model system, we demonstrated a large variation in net intestinal absorption from the apical to the basolateral side. Especially dasatinib, erlotinib, crizotinib, and afatinib showed a highly negative ratio because of either a poor apical-basolateral transfer (crizotinib, erlotinib, and afatinib) or a high reversed basolateral/apical reflux to the apical (gut) side) (dasatinib and crizotinib). Sorafenib, sunitinib, and gefitinib showed a relatively good absorption. Despite these negative properties, the former drugs show clinical efficacy even against solid tumors, although dasatinib has only shown efficacy against multiple myeloma. This might be related to a rapid accumulation in blood compartments such as red blood cells, enabling delivery to the site of action.

New TKIs are often selected based on their high inhibition of potential targets, which is one of the essential features for drug development. However, tumors may prove to be resistant to these drugs (a pharmacokinetic resistance) because of a poor pharmacokinetic behavior; due to a poor absorption from the gut, they do not reach the tumor, while they may be excellent substrates for efflux pumps present on tumor cells. It is, therefore, suggested that absorption properties of new potential TKIs should be investigated carefully, along with physicochemical properties to predict their absorption behavior or substrate specificity for efflux pumps.

## Figures and Tables

**Table 1:** Properties of tyrosine kinase inhibitors.

Drug	Targets	Disease	Dose	Effect of Food (%)	Protein (%)	Steady State Concentration (µM)	Vd (l)	t½ (h)	Bioavailability (%)
Gefitinib [3,4,5,6]	EGFR + mutation	NSCLC	250 mg daily	~ 37% increase [7]	~90	0.11–0.33	1700	32	35–60 [8,9]
Erlotinib [8,10,11,12,13]	EGFR + mutation	NSCLC, pancreatic cancer	150 mg daily	~ 40% increase [14]	~95	2.54–5.08	232	15–30	76 [15]
Afatinib [16]	EGFR + mutation	NSCLC	40 mg daily	~ 50% decrease	~95	0.062–0.16	2870	36.6	No data available
Sunitinib [17]	VEGFR, KDR, FLT3	GIST, RCC, HCC, pNET	50 mg daily	~12% increase [18]	~98	0.15–0.25	1900	25–60	39
Sorafenib [19,20,21] ^1^	KDR, Ras/Raf pathway	HCC, RCC, DTC	100–600 mg BID	Inconsistent ~ 29% decrease ~14% increase [22,23]	~95	6.28–23.45	212	22–28	Estimated 38–49%
Crizotinib [24,25,26]	ALK, c-MET	NSCLC	250 mg daily	~ 14% decrease	~ 90%	0.22–0.44	4200–4900	43–51	43 [27]
Dasatinib [19,28,29,30]	BCR-Abl	CML	100–140 mg daily	~ 14% increase [31]	~ 94%	0.19–0.27	2500	3–4	14–27 [32] (animal)

^1^ Where CML is chronic myeloid leukemia; DTC, differentiated thyroid carcinoma; FLT3, fms like tyrosine kinase 3; GIST, gastro-intestinal stromal tumors; HCC, hepatocellular carcinoma; KDR, Kinase insert domain receptor; pNET, pancreatic neuroendocrine tumors’; RCC, renal cell carcinoma; Vd, volume of distribution; BID, *bis in die* (twice a day). Protein indicates the percent drug that is protein bound.

**Table 2:** Physical and transport properties of seven approved tyrosine kinase inhibitors.

Drug	µM	P*app*_(×10_^−6^_)_ cm/s (Apical Basolateral)	P*app*_(×10_^−6^_)_ cm/s (Basolateral Apical)	Efflux Ratio	Log P	Log D (pH 5.5)	Log D (pH 7.5)
Gefitinib	20	0.38 ± 0.05	0.39 ± 0.038	1.0	4.11	1.96	3.55
	10	0.57 ± 0.43	0.19 ± 0.12	0.3			
Erlotinib	20	0.13 ± 0.001	0.70 ± 0.30	5.4	2.39	2.74	3.04
	10	0.48 ± 0.26	0.79 ± 0.65	1.6			
with sorafenib	10	0.35 ± 0.17	0.48 ± 0.30	1.4			
Afatinib	10	1.5 ± 2.2	10.9 ± 5.0	7.3	3.59	1.00	2.87
Sunitinib	20	7.7 ± 4.6	12.6 ± 12.3	1.6	3.15	−0.09	1.09
Sorafenib	20	14.1 ± 11.5	11.4 ± 5.6	0.81	5.16	4.26	4.26
	10	10.1 ± 9.5	9.3 ± 5.8	0.92			
with Erlotinib	10	18.6 ± 13.2	13.3 ± 10.5	0.71			
Crizotinib	10	Unmeasurable	7.7 ± 5.5	>7 *	4.73	0.30	1.80
Dasatinib	20	3.4 ± 3.4	20.4 ± 13.5	6.0	2.24	−0.44	2.17

Values are means ± SD from at least 3 separate experiments. Crizotinib apical to basal lateral values were undetectable at the concentration of 10 µM, therefore, * the efflux ratio only can be estimated to be greater than 7 if a P*app* A–B of 1 is assumed.

## Supplementary Materials

The following are available online at https://www.mdpi.com/2072-6694/12/11/3322/s1, Figure S1: Schematic representation of the CaCo2 Transwell system, Figure S2: Accumulation of crizotinib and sunitinib in MCF/MR breast cancer cells and T98 and U251 glioma cells; effect of bafilomycin.
