# Supplementary material for: Epithelial Transfer of the Tyrosine Kinase Inhibitors Erlotinib, Gefitinib, Afatinib, Crizotinib, Sorafenib, Sunitinib, and Dasatinib: Implications for Clinical Resistance
Source: Cancers (Basel). 2020 Nov 10;12(11):3322. doi: 10.3390/cancers12113322 (PMC7696666; doi:10.3390/cancers12113322)
Supplement: Supplementary file 1 [file cancers-12-03322-s001.pdf]

Supplementary Materials

Epithelial Transfer of the Tyrosine Kinase Inhibitors Erlotinib, Gefitinib, Afatinib, Crizotinib, Sorafenib, Sunitinib and Dasatinib: Implications for Clinical Resistance

Richard J. Honeywell, Ietje Kathmann, Elisa Giovannetti, Carmelo Tibaldi, Egbert F. Smit, Maria N. Rovithi, Henk M.W. Verheul and Godefridus J. Peters

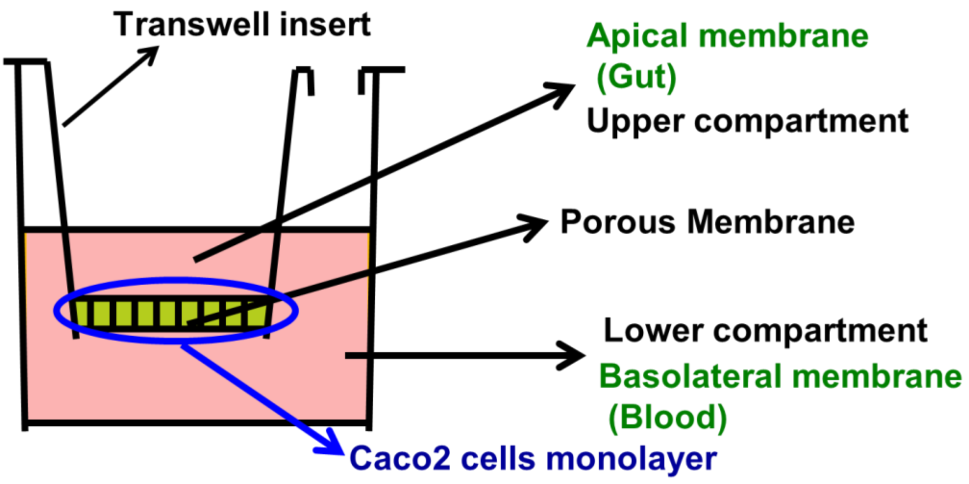

Figure S1. Schematic representation of the CaCo-2 transwell system. CaCo2 cells were established as a monolayer using the BIOCOAT® HTS system. Cells were plated in the insert after which they polarized with the upper side being the donor compartment (apical side, A) and the lower side the receiver compartment (basolateral side, B). Using a Trans Epithelial Electrical Resistance (TEER) meter (Millicell®-ERS, Millipore) the electrical resistance was measured as an indication for the integrity of the transwell. Only wells with a value between 600–1600 Ω cm^2 were used according to the specification of Becton Dickinson BIOCOAT. Drugs were added to either the apical or the basolateral side as indicated in the experiments. At certain time-points 50 μL samples were taken for analysis.

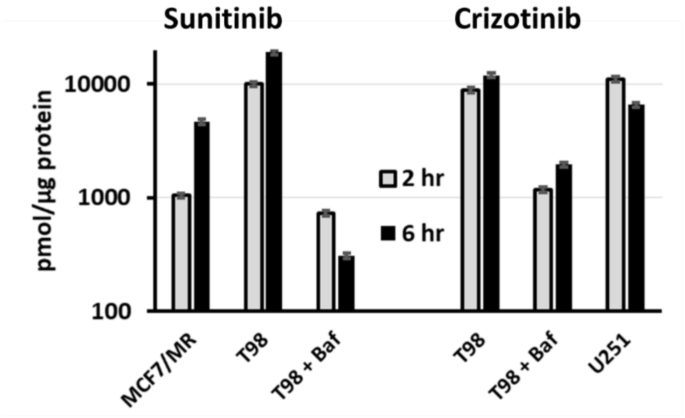

Figure S2. Accumulation of crizotinib and sunitinib in MCF/MR breast cancer cells and T98 and U251 glioma cells; effect of bafilomycin. Intracellular accumulation of sunitinib and crizotinib was measured after exposure to 5 µM in the presence or absence of 50 nM bafilomycin (Baf) and was performed similar to the experiments with CaCo2, WiDr and HT29 monolayer cells. Values are means ± SEM of 3 separate experiments. MCF7/MR is a variant of MCF7 breast cancer cells which is resistant to mitoxantrone and has an overexpression of the ATP Binding Cassette (ABC) efflux transporter ABCG2 (BCRP).

© 2020 by the authors. Licensee MDPI, Basel, Switzerland. This article is an open access article distributed under the terms and conditions of the Creative Commons Attribution (CC BY) license (http://creativecommons.org/licenses/by/4.0/).
